# Predictive models of long-term anatomic outcome in age-related macular degeneration treated with as-needed Ranibizumab

**DOI:** 10.1186/s12886-017-0544-x

**Published:** 2017-08-18

**Authors:** Lucia Gonzalez-Buendia, Santiago Delgado-Tirado, M. Rosa Sanabria, Itziar Fernandez, Rosa M. Coco

**Affiliations:** 1 Instituto de Oftalmobiologia Aplicada (IOBA), Campus Miguel Delibes, University of Valladolid, P° de Belén n° 17, 47011 Valladolid, Spain; 2 Clinic University Hospital of Valladolid, Valladolid, Spain; 3 Health Complex of Palencia, Palencia, Spain; 4 Ciber BBN, Zaragoza, Spain

**Keywords:** Age-related macular degeneration, Choroidal neovascularization, Anti-VEGF

## Abstract

**Background:**

To analyze predictors and develop predictive models of anatomic outcome in neovascular age-related macular degeneration (AMD) treated with as-needed ranibizumab after 4 years of follow-up.

**Methods:**

A multicenter consecutive case series non-interventional study was performed. Clinical, funduscopic and OCT characteristics of 194 treatment-naïve patients with AMD treated with as-needed ranibizumab for at least 2 years and up to 4 years were analyzed at baseline, 3 months and each year until the end of the follow-up. Baseline demographic and angiographic characteristics were also evaluated. R Statistical Software was used for statistical analysis. Main outcome measure was final anatomic status.

**Results:**

Factors associated with less probability of preserved macula were diagnosis in 2009, older age, worse vision, presence of atrophy/fibrosis, pigment epithelium detachment, and geographic atrophy/fibrotic scar/neovascular AMD in the fellow eye. Factors associated with higher probability of GA were presence of atrophy and greater number of injections, whereas male sex, worse vision, lesser change in central macular thickness and presence of fibrosis were associated with less probability of GA as final macular status. Predictive model of preserved macula vs. GA/fibrotic scar showed sensibility of 77.78% and specificity of 69.09%. Predictive model of GA vs. fibrotic scar showed sensibility of 68.89% and specificity of 72.22%.

**Conclusions:**

We identified predictors of final macular status, and developed two predictive models. Predictive models that we propose are based on easily harvested variables, and, if validated, could be a useful tool for individual patient management and clinical research studies.

## Background

Age-related macular degeneration (AMD) is the major cause of visual impairment in developed countries in people over 60 years [1]. Currently, almost every patient showing active neovascular AMD undergoes treatment with drugs targeting vascular endothelial growth factor (VEGF), as these drugs can slow progression of this form of the disease [2]. Ranibizumab, an anti-VEGF agent, is widely used for the management of choroidal neovascularization (CNV) secondary to AMD. Anti-VEGF agents have shown rare complications associated to its use, nevertheless, recent reports describe vision-threatening events noted during follow-up of eyes receiving these treatments [3]. These events include development of geographic atrophy (GA) [4] and fibrotic scar formation [5].

The area of a CNV lesion often develops retinal pigment epithelium (RPE) and choriocapillary atrophy. These atrophic lesions are clinically indistinguishable from de novo GA [6]. Fibrotic scar formation within the retina or the subretinal space occurs in the natural course of neovascular AMD [7], and has been identified as an important cause of visual loss after treatment with anti-VEGF agents [3]. However, factors associated with GA and fibrotic scar formation after treatment with ranibizumab remain to be elucidated.

Our study aims to investigate predictive factors associated to long-term anatomic outcome in patients with AMD after as-needed treatment with ranibizumab, including data collected from seven centres in the Spanish region of Castilla & León. The identification of these factors may provide helpful information to predict final macular status of patients with AMD receiving this regimen of ranibizumab. Additionally, basing on these variables, we propose predictive models of anatomic outcome.

## Methods

We performed an observational consecutive case series study in seven centres in Castilla & León, Spain. A systematic review of medical charts of patients with AMD treated with ranibizumab (Lucentis, Genentech, Inc., South San Francisco, CA), collected in our database was done. The study was designed to survey longstanding funduscopic outcome. Descriptive results of the whole sample have already been published [8].

Protocol was approved by the ethical committee of the coordinating centre (Research and Ethics Commission of IOBA Eye Institute, University of Valladolid, Valladolid, Spain) and of each participant institution. The study was conducted in compliance with the guidelines in the Declaration of Helsinki. Written informed consent was obtained from all participants.

### Patients

The study included treatment-naïve neovascular AMD patients who started treatment between January 1, 2008 and December 31, 2012, following as-needed regimen of injections, with at least 2-years of follow-up. Only one eye of each patient was enrolled; when both eyes met inclusion criteria, the eye with longer follow-up was enrolled, and selection was made randomly if both had the same follow-up period. Patients suffering any other associated sight-threatening pathology (except for cataract), late baseline AMD, patients who did not complete at least 24 months of follow-up, those who discontinued treatment for any reason during follow-up, and those who showed no response to treatment (no morphologic nor functional improvement after 3 ranibizumab injections) were excluded.

### Variables

Baseline characteristics recorded are shown in Table 1. Total time of follow-up was also noted. Snellen distance best-corrected visual acuity (BCVA), OCT assessment, presence of macular-associated lesions and number of visits and injections were recorded at baseline, 3 months and at 1, 2, 3 and 4 years of follow-up.

**Table 1:** Baseline characteristics of patients

	Preserved vs. GA/fibrotic scar	GA vs. fibrotic scar
Characteristics	*n* = 194	*n* = 153
Mean age (years)	78.01	78.68
Sex (n)		
Male	78	89
Female	116	64
Affected eye (n)		
Right	101	88
Left	93	65
Year of diagnosis (n)		
2007	4	3
2008	49	39
2009	101	85
2010	40	26
Delay of treatment (n)		
< 30 days	128	99
(30–90] days	50	43
> 90 days	16	11
Angiographic type of lesion (n)		
Classic	43	36
Predominantly classic	13	11
Minimally classic	14	13
Occult	62	45
Others	3	2
Status of the fellow eye (n)		
Initial/intermediate AMD	75	54
Neovascular	13	9
Atrophic	38	36
Disciform scar	37	30
Others	21	15
Status of the lens (n)		
Cataract	108	88
Pseudophakia	53	40
Transparent	7	3

*GA* geographic atrophy, *AMD* age-related macular degeneration

Macular associated lesions were assessed by colour fundus photographs, fluorescein angiography (FA), fundus autofluorescence and OCT. These lesions were identified as bleeding >50%, presence of pigment epithelium detachment (PED), RPE tear, retinal angiomatous proliferation (RAP), polypoidal vasculopathy and presence of a minimum area of fibrosis or atrophy. These two latter lesions never involved fovea and were not the main component of lesion. Snellen distance BCVA was transformed into the logarithm of the minimum angle of resolution (logMAR), using a validated procedure [9]. OCT morphology data (presence or absence of subretinal fluid and/or thickening >100 μm compared to the previous visit, (both or none) was also gathered. Central macular thickness change was evaluated following a procedure previously described [8].

Final funduscopic status of the studied eye was classified as active, inactive with predominantly fibrotic disciform scar, inactive with predominantly atrophic scar and inactive well-preserved macula, and the rest were excluded of the study. To assess predictive factors and models of anatomic outcome, only eyes with final fibrotic scar, atrophy or well-preserved macula at the end of the study were analysed. To classify funduscopic results of contralateral eye, International ARM classification was used [10].

### Statistical analysis

Quantitative characteristics were expressed as mean ± standard deviation (SD), and qualitative variables were described in percentages. Statistical analysis was performed using R Statistical Software (R Core Team; Vienna, Austria) [11]. To avoid influence of missing data we used the Copy Mean method [12], implemented at the Package Longitudinal Data of R (Longitudinal Data. R package version 2.2).

Depending on the main variable assessed, the statistical analysis was divided in two sections. First, probability of macular preservation was compared to probability of either GA or fibrotic scar. For the second part of the assessment, eyes showing well-preserved macula as final anatomic status were excluded, and probability of GA at the end of the follow-up was compared to probability of fibrotic scar.

A binary logistic regression model was fitted to identify potential predictors of functional outcome between collected variables. Estimated odds ratio (OR) was used to quantify the importance of each potential predictor. Variables with a univariate *p* value lower than 0.1 were identified as relevant predictors. Then, we performed a multivariate logistic regression model based on the best set of relevant predictors according to Akaike Information Criteria [13]. The bestglm package [14] was used to enumerate and evaluate all possible models. Inter-correlation between the finally selected predictors was evaluated using the Variance Inflation Factor implemented in the car package [15]. A Variance Inflation Factor value higher than 5 indicated presence of multicollinearity.

In order to assess the performance of prediction model, a leave-one-out cross-validation process was used for internal validation. Three aspects were evaluated: precision, calibration and discrimination ability.

The Brier Score was used as global measure of the precision [16]. This score is based upon individual differences between predicted risks in terms of likelihood and observed final outcomes. The Brier score ranges, from 0 for a perfect degree of agreement to 1 for the worst possible degree of agreement.

To evaluate the calibration of the model, two measures were used: the calibration-in-the large, that, in a perfectly calibrated model will be 0, and the Calibration Slope that in such model will be 1. We also used the Hosmer-Lemeshow test, which is significant for badly calibrated models [17].

Receiver Operating Characteristic (ROC) curve analysis was used to assess the discrimination ability of the fitted models. They were evaluated and compared according to the area under the ROC curve (AUC). In addition, the sensitivity and specificity for the ROC curve was obtained by setting an optimal threshold using the pROC package [18].

## Results

Notes from 1236 patients treated with anti-VEGF drugs at seven hospitals were evaluated, and 314 eyes were identified as eligible [8]. Nevertheless, for this part of the study 61 eyes that had discontinued treatment were excluded, and so were those with active or unclassifiable macular anatomic status, thus, 194 eyes (194 patients, 112 women and 82 men) were analysed. Baseline characteristics of the sample appear in Table 1.

First, to study predictive factors and models of probability of well-preserved macula, data from 194 eyes presenting late AMD were used, from which 153 showed either GA/fibrotic scar and 41 showed well-preserved macula. Afterwards, a second study was performed with a sample of 153 eyes, those 153 with GA/scar, from which 72 showed GA and 81 fibrotic scar.

### Preserved vs. GA/fibrotic scar

To determine factors associated to anatomic preservation versus developing GA or fibrotic scar, a subset of 194 eyes from 194 patients (116 women, 78 men) were included. Mean age was 78.01 years (range 55–93; SD 7.55). Mean follow-up was 98.03 months (range 24.3–161.5; SD 28.08; median 98.25). After 4 years of follow-up, 41 eyes presented preserved macular anatomy (21.13%) and 153 presented GA or fibrotic scar (78.87%).

Results of univariate analysis appear in Table 2. Statistically significant higher probability of preserved macular anatomy was found among patients with transparent lens. Contrarily, negative predictors of preserved macular anatomy, were diagnosis in 2009, older age, worse BCVA in all visits, PED at 1 and 2 years, small area of atrophy (not involving the fovea nor being the main component of lesion) at 1 and 4 years of follow-up, small area of fibrosis (not involving the fovea nor being the main component of lesion) at 2, 3 and 4 years, atrophic AMD in the fellow eye at baseline and at the end of the follow-up, neovascular AMD in the fellow eye at the end of the follow-up, and fibrotic scar in the fellow eye at the end of the follow-up.

**Table 2:** Univariate analysis for preserved vs. geographic atrophy/fibrotic scar

	Baseline					3 months				
	n (%)	OR	CI 95%		*p*-value	n (%)	OR	IC 95%		*p*-value
Age	194	*0.9473*	*0.9058*	*0.9907*	*0.018*	-	-	-	-	-
Sex										
Female	116 (59.79)	1	-	-	-	-	-	-	-	-
Male	78 (40.21)	0.7211	0.3506	0.1.4829	0.374	-	-	-	-	-
Year of diagnosis										
2007	4 (2.06)	0.619	0.0588	6.5209	0.6897	-	-	-	-	-
2008	49 (25.26)	0.4762	0.1839	1.2328	0.1263	-	-	-	-	-
2009	101 (52.06)	*0.3496*	*0.1508*	*0.8106*	*0.0143*	-	-	-	-	-
2010	40 (20.62)	1	-	-	-	-	-	-	-	-
Delay of treatment										
< 30 days	128 (65.98)	1	-	-	-	-	-	-	-	-
(30–90] days	50 (25.77)	0.5557	0.226	1.3664	0.2006	-	-	-	-	-
> 90 days	16 (8.25)	1.5517	0.4986	4.8289	0.4481	-	-	-	-	-
Angiographic type of lesion										
Classic	43 (31.85)	0.5147	0.1925	1.3759	0.1855	-	-	-	-	-
Predominantly classic	13 (9.63)	0.4813	0.0965	2.3997	0.3723	-	-	-	-	-
Minimally classic	14 (10.37)	0.2036	0.0247	1.678	0.1391	-	-	-	-	-
Occult	62 (45.93)	1	-	-	-	-	-	-	-	-
Others	3 (2.22)	1.3235	0.1126	15.5608	0.8236	-	-	-	-	-
Status of the fellow eye										
Initial/intermediate AMD	75 (40.76)	1	-	-	-	-	-	-	-	-
Neovascular	13 (7.07)	1.1429	0.3174	4.115	0.8381	-	-	-	-	-
Atrophic	38 (20.65)	*0.1429*	*0.0315*	*0.6469*	*0.0116*	-	-	-	-	-
Disciform scar	37 (20.11)	0.6	0.2286	1.5746	0.2994	-	-	-	-	-
Others	21 (11.41)	1.0286	0.3519	3.0064	0.9589	-	-	-	-	-
Status of the lens										
Cataract	108 (64.29)	1	-	-	-	-	-	-	-	-
Pseudophakia	53 (31.55)	1.43	0.6477	3.1572	0.3761	-	-	-	-	-
Transparent	7 (4.17)	*5.8667*	*1.216*	*28.3039*	*0.0276*	-	-	-	-	-
BCVA	194	*0.1296*	*0.0477*	*0.3517*	*0.0001*	194	*0.0668*	*0.0194*	*0.2301*	*<0.0001*
CMT change	-	-	-	-	-	191	0.8438	0.2975	2.3933	0.7496
OCT assessment										
SRF	49 (28)	0.4263	0.0917	1.9823	0.2769	60 (31.91)	1.3636	0.6457	2.8796	0.4161
Thickening	21 (12)	4.05	0.5372	30.5344	0.1748	7 (3.72)	-	-	-	0.9867
Both	101 (57.71)	1	-	-	-	9 (4.79)	0.5114	0.0607	4.3051	0.5372
None	4 (2.29)	1.7868	0.819	3.8982	0.1448	112 (59.57)	1	-	-	-
Macular associated lesions										
Bleeding >50%	20 (14.6)	0.402	0.1046	1.5454	0.1847	5 (4.24)	0.6964	0.0716	6.7734	0.7553
PED	37 (27.01)	0.3559	0.1193	1.0621	0.064	23 (19.49)	0.2653	0.055	1.28	0.0984
RPE tear	2 (1.46)	-	-	-	0.9969	3 (2.54)	-	-	-	0.9976
Initial minimal atrophy	5 (3.65)	-	-	-	0.9951	8 (6.78)	-	-	-	0.9961
Initial minimal fibrosis	10 (7.3)	-	-	-	0.9931	23 (19.49)	-	-	-	0.9934
RAP	2 (1.46)	2.2778	0.1349	38.4694	0.5681	2 (1.69)	2.7857	0.163	47.597	0.4793
PV	2 (1.46)	-	-	-	0.9969	1 (0.85)	-	-	-	0.9985
Others	59 (43.07)	-	-	-	-	53 (44.92)	-	-	-	-
Total time of treatment	-	-	-	-	-	-	-	-	-	-
Number of injections	-	-	-	-	-	194	1.9616	0.9564	4.0233	0.066
Number of visits	-	-	-	-	-	170	1.4041	0.8748	2.2536	0.1598
	1 year					2 years				
	n (%)	OR	CI 95%		*p*-value	n (%)	OR	CI 95%		*p*-value
Age	-	-	-	-	-	-	-	-	-	-
Sex										
Female	-	-	-	-	-	-	-	-	-	-
Male	-	-	-	-	-	-	-	-	-	-
Year of diagnosis										
2007	-	-	-	-	-	-	-	-	-	-
2008	-	-	-	-	-	-	-	-	-	-
2009	-	-	-	-	-	-	-	-	-	-
2010	-	-	-	-	-	-	-	-	-	-
Delay of treatment										
< 30 days	-	-	-	-	-	-	-	-	-	-
(30–90] days	-	-	-	-	-	-	-	-	-	-
> 90 days	-	-	-	-	-	-	-	-	-	-
Angiographic type of lesion										
Classic	-	-	-	-	-	-	-	-	-	-
Predominantly classic	-	-	-	-	-	-	-	-	-	-
Minimally classic	-	-	-	-	-	-	-	-	-	-
Occult	-	-	-	-	-	-	-	-	-	-
Others	-	-	-	-	-	-	-	-	-	-
Status of the fellow eye										
Initial/intermediate AMD	-	-	-	-	-	-	-	-	-	-
Neovascular	-	-	-	-	-	-	-	-	-	-
Atrophic	-	-	-	-	-	-	-	-	-	-
Disciform	-	-	-	-	-	-	-	-	-	-
Others	-	-	-	-	-	-	-	-	-	-
Status of the fellow eye										
Cataract	-	-	-	-	-	-	-	-	-	-
Pseudophakia	-	-	-	-	-	-	-	-	-	-
Transparent	-	-	-	-	-	-	-	-	-	-
BCVA	194	*0.0227*	*0.0058*	*0.0891*	*<0.0001*	194	*0.0694*	*0.026*	*0.1851*	*<0.0001*
CMT change	192	0.5216	0.1953	1.3932	0.1941	192	1.3015	0.5533	3.0618	0.546
OCT assessment										
SRF	52 (27.96)	0.6667	0.2882	1.542	0.2769	33 (18.33)	1.5	0.6232	3.6105	0.3656
Thickening	11 (5.91)	0.7078	0.1441	3.478	0.1748	6 (3.33)	2	0.3472	11.52	0.4378
Both	10 (5.38)	0.3539	0.0429	2.9208	-	11 (6.11)	0.8889	0.181	4.3646	0.8847
None	113 (60.75)	1	-	-	0.1448	130 (72.22)	1	-	-	-
Macular associated lesions										
Bleeding >50%	1 (0.76)	-	-	.	0.9986	1 (0.75)	-	-	-	0.9986
PED	17 (12.88)	*0.0882*	*0.0107*	*0.7305*	*0.0244*	18 (13.43)	*0.2267*	*0.0547*	*0.9385*	*0.0406*
RPE tear	4 (3.03)	0.4706	0.045	4.9191	0.529	2 (1.49)	-	-	-	0.998
Initial minimal atrophy	22 (16.67)	*0.1412*	*0.0291*	*0.6859*	*0.0152*	26 (19.4)	-	-	-	0.9926
Initial minimal fibrosis	47 (33.61)	-	-	-	0.9902	54 (40.3)	*0.0436*	*0.009*	*0.2103*	*0.0001*
RAP	0 (0)	-	-	-	-	0 (0)	-	-	-	-
PV	0 (0)	-	-	-	-	1 (0.75)	-	-	-	0.9986
Others	41 (31.06)	1	-	-	-	32 (23.88)	1	-	-	-
Total time of treatment	-	-	-	-	-	-	-	-	-	-
Number of injections	194	1.2133	0.9583	1.5362	-	189	1.1721	0.9163	1.4992	0.2062
Number of visits	184	1.1135	0.9391	1.3202	-	187	1.0613	0.9327	1.2076	0.3666
	3 years					4 years				
	n (%)	OR	CI 95%		*p*-value	n (%)	OR	CI 95%		*p*-value
Age	-	-	-	-	-	-	-	-	-	-
Sex										
Female	-	-	-	-	-	-	-	-	-	-
Male	-	-	-	-	-	-	-	-	-	-
Year of diagnosis										
2007	-	-	-	-	-	-	-	-	-	-
2008	-	-	-	-	-	-	-	-	-	-
2009	-	-	-	-	-	-	-	-	-	-
2010	-	-	-	-	-	-	-	-	-	-
Delay of treatment										
< 30 days	-	-	-	-	-	-	-	-	-	-
(30–90] days	-	-	-	-	-	-	-	-	-	-
> 90 days	-	-	-	-	-	-	-	-	-	-
Angiographic type of lesion										
Classic	-	-	-	-	-	-	-	-	-	-
Predominantly classic	-	-	-	-	-	-	-	-	-	-
Minimally classic	-	-	-	-	-	-	-	-	-	-
Occult	-	-	-	-	-	-	-	-	-	-
Others	-	-	-	-	-	-	-	-	-	-
Status of the fellow eye										
Initial/intermediate AMD	-	-	-	-	-	50 (27.17)	1	-	-	-
Neovascular	-	-	-	-	-	21 (11.41)	*0.075*	*0.0093*	*0.6042*	*0.015*
Atrophic	-	-	-	-	-	50 (27.17)	*0.1304*	*0.0406*	*0.4194*	*0.0006*
Disciform scar	-	-	-	-	-	44 (23.91)	*0.3857*	*0.1529*	*0.9733*	*0.0437*
Others	-	-	-	-	-	19 (10.33)	0.6923	0.2257	2.1232	0.5201
Status of the lens										
Cataract	-	-	-	-	-	-	-	-	-	-
Pseudophakia	-	-	-	-	-	-	-	-	-	-
Transparent	-	-	-	-	-	-	-	-	-	-
BCVA	194	*0.0523*	*0.0182*	*0.1501*	*<0.0001*	194	*0.0595*	*0.0219*	*0.1616*	*<0.0001*
CMT change	192	1.2116	0.5475	2.6811	0.6358	192	1.3213	0.6428	2.7159	0.4485
OCT assessment										
SRF	19 (14.62)	0.9576	0.2885	3.1789	0.9435	5 (7.68)	-	-	-	0.9926
Thickening	5 (3.85)	0.8977	0.0954	8.4467	0.9248	1 (1.52)	-	-	-	0.9962
Both	5 (3.85)	5.3864	0.8465	34.2744	0.0745	3 (4.55)	1.5357	0.1292	18.248	0.7341
None	101 (77.69)	1	-	-	-	57 (86.36)	1	-	-	-
Macular associated lesions										
Bleeding >50%	0 (0)	-	-	-	-	0 (0)	-	-	-	-
PED	9 (8.65)	0.1136	0.012	1.0764	0.058	1 (1.82)	-	-	-	0.9963
RPE tear	3 (2.28)	-	-	-	0.9975	1 (1.82)	-	-	-	0.9963
Initial minimal atrophy	26 (25)	-	-	-	0.9926	13 (23.64)	*0.0417*	*0.0035*	*0.4908*	*0.0115*
Initial minimal fibrosis	45 (43.27)	*0.0423*	*0.0081*	*0.2216*	*0.0002*	31 (56.36)	*0.0167*	*0.0015*	*0.1887*	*0.0009*
RAP	0 (0)	-	-	-	-	0 (0)	-	-	-	-
PV	0 (0)	-	-	-	-	0 (0)	-	-	-	-
Others	21 (20.19)	1	-	-	-	9 (16.36)	1	-	-	-
Total time of treatment	-	-	-	-	-	194	1.0131	0.982	1.0453	0.4134
Number of injections	147	1.0238	0.8646	1.2122	0.7852	71	1.0032	0.8306	1.2116	0.9738
Number of visits	147	1.1359	0.9571	1.3482	0.1448	76	0.9568	0.7498	1.2211	0.7229

A total of 194 patients were included in the final univariate analysis for presence of preserved macula vs. geographic atrophy/fibrotic scar. This table shows all the variables evaluated in each visit during the 4 years of follow-up. OR: odds ratio, CI: confidence interval, AMD: age-related macular degeneration, BCVA: best corrected visual acuity, CMT: central macular thickness, SRF: subretinal fluid, PED: pigment epithelium detachment, RPE: retinal pigment epithelium, RAP: retinal angiomatous proliferation, PV: polypoidal vasculopathy. Statistically significant results appear in italic

**Fig. 1:**
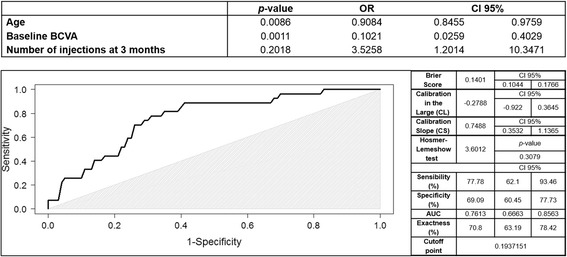
Multivariate analysis and predictive model Receiver Operating Characteristic (ROC) curve of preserved macula vs. geographic atrophy/fibrotic scar. The upper chart shows results of multivariate analysis. The figure below shows ROC curve of the predictive model. The area under the curve reveals that this model has a good reliability in the prediction of final macular anatomic status. The greyish area indicates where the model loses its reliability. Besides, on the right internal validation data is shown. CI: confidence interval, AUC: area under the curve, CL: calibration in the large, CS: calibration slope

Then, multivariate analysis (Fig. 1) to find the best predictive model was performed using those previous variables with *p* < 0.1 and measurements recorded after the first year of follow-up were excluded, as they would have low predictive value. Thus, diagnosis in 2009, age, baseline BCVA, PED at baseline, number of injections at 3 months and presence of atrophy within the fellow eye at baseline were considered.

The best-fitted model found (Fig. 1) included 3 variables: age, baseline BCVA and number of injections at 3 months.

### Geographic atrophy vs. fibrotic scar

A subset of 153 eyes from 153 patients (89 women, 62 men) was analysed for this purpose. Mean age was 78.68 years (range 55–93; SD 7.19). Mean follow-up was 97.24 months (range 24.3–161.5; SD 27.3; median 98). After 4 years of follow-up, 72 eyes presented GA (47.06%) whereas 81 eyes presented fibrotic scar (52.94%). Results of univariate analysis are shown in Table 3.

**Table 3:** Univariate analysis for geographic atrophy vs. fibrotic scar

	Baseline					3 months				
	n (%)	OR	CI 95%		*p*-value	n (%)	OR	CI 95%		*p*-value
Age	153	1.0119	0.9678	1.058	0.6024	-	-	-	-	-
Sex										
Female	89 (58.17)	1	-	-	-	-	-	-	-	-
Male	64 (41.83)	*0.4579*	*0.2368*	*0.8857*	*0.0203*	-	-	-	-	-
Year of diagnosis										
2007	3 (1.96)	0.9444	0.075	11.8891	0.9647	-	-	-	-	-
2008	39 (25.49)	1.314	0.4693	3.6789	0.6031	-	-	-	-	-
2009	85 (55.56)	2.2279	0.8935	5.5553	0.0857	-	-	-	-	-
2010	26 (16.99)	1	-	-	-	-	-	-	-	-
Delay of treatment										
< 30 days	99 (64.71)	1	-	-	-	-	-	-	-	-
(30–90] days	43 (28.1)	0.8411	0.4097	1.7271	0.6374	-	-	-	-	-
> 90 days	11 (7.19)	0.8854	0.2535	3.0921	0.8487	-	-	-	-	-
Angiographic type of lesion										
Classic	36 (33.64)	1.1957	0.4961	2.8816	0.6905	-	-	-	-	-
Predominantly classic	11 (10.28)	0.3587	0.0841	1.5291	0.1658	-	-	-	-	-
Minimally classic	13 (12.15)	0.5978	0.1694	2.1097	0.4239	-	-	-	-	-
Occult	45 (42.06)	1	-	-	-	-	-	-	-	-
Others	2 (1.87)	-	-	-	0.9879	-	-	-	-	-
Status of the fellow eye										
Initial/intermediate AMD	54 (37.5)	1	-	-	-	-	-	-	-	-
Neovascular	9 (6.25)	4.375	0.8314	23.0235	0.1015	-	-	-	-	-
Atrophic	36 (25)	0.8929	0.3806	2.0947	0.7945	-	-	-	-	-
Disciform scar	30 (20.83)	1.25	0.511	3.0579	0.6249	-	-	-	-	-
Others	15 (10.42)	0.625	0.1882	2.0755	0.4428	-	-	-	-	-
Status of the lens										
Cataract	88 (67.18)	1	-	-	-	-	-	-	-	-
Pseudophakia	40 (30.53)	1.1679	0.5516	2.4728	0.6851	-	-	-	-	-
Transparent	3 (2.29)	1.9111	0.1671	21.8512	0.6024	-	-	-	-	-
BCVA	153	0.8892	0.4752	1.6636	0.7132	153	1.0638	0.5598	2.0217	0.8502
CMT change	-	-	-	-	-	150	1.4185	0.5473	3.677	0.4718
OCT assessment										
SRF	34 (25)	1.25	0.5603	2.7889	0.5858	45 (30)	1.4286	0.6966	2.9297	0.3304
Thickening	19 (13.97)	0.9091	0.3309	2.4976	0.8534	7 (4.67)	1.6667	0.3525	7.8809	0.5193
Both	81 (59.56)	1	-	-	-	8 (5.33)	0.1786	0.0211	1.5119	0.1139
None	2 (1.47)	1.25	0.0755	20.6845	0.8762	90 (60)	1	-	-	-
Macular associated lesions										
Bleeding >50%	17 (15.45)	0.8466	0.2729	2.6266	0.7731	4 (4.04)	0.2857	0.0273	2.9932	0.2959
PED	32 (29.09)	1.2245	0.4837	3.1	0.6691	21 (21.21)	0.6429	0.2207	1.8728	0.418
RPE tear	2 (1.82)	-	-	-	0.9922	3 (3.03)	1.7143	0.1433	20.504	0.6703
Initial minimal atrophy	5 (4.55)	1.4286	0.2156	9.4665	0.7119	8 (8.08)	2.5714	0.4606	14.355	0.2817
Initial minimal fibrosis	10 (9.09)	0.2381	0.045	1.2599	0.0914	23 (23.23)	*0.3025*	*0.0983*	*0.9306*	*0.037*
RAP	1 (0.91)	-	-	-	0.9945	1 (1.01)	-	-	-	0.9914
PV	2 (1.82)	-	-	-	0.9922	0 (0)	-	-	-	-
Others	41 (37.27)	1	-	-	-	39 (39.39)	1	-	-	-
Total time of treatment	-	-	-	-	-	-	-	-	-	-
Number of injections	-	-	-	-	-	153	*2.5099*	*1.318*	*4.7796*	*0.0051*
Number of visits	-	-	-	-	-	133	0.9856	0.6336	1.5332	0.9486
	1 year					2 years				
	n (%)	OR	CI 95%		*p*-value	n (%)	OR	CI 95%		*p*-value
Age	-	-	-	-	-	-	-	-	-	-
Sex										
Female	-	-	-	-	-	-	-	-	-	-
Male	-	-	-	-	-	-	-	-	-	-
Year of diagnosis										
2007	-	-	-	-	-	-	-	-	-	-
2008	-	-	-	-	-	-	-	-	-	-
2009	-	-	-	-	-	-	-	-	-	-
2010	-	-	-	-	-	-	-	-	-	-
Delay of treatment										
< 30 days	-	-	-	-	-	-	-	-	-	-
(30–90] days	-	-	-	-	-	-	-	-	-	-
> 90 days	-	-	-	-	-	-	-	-	-	-
Angiographic type of lesion										
Classic	-	-	-	-	-	-	-	-	-	-
Predominantly classic	-	-	-	-	-	-	-	-	-	-
Minimally classic	-	-	-	-	-	-	-	-	-	-
Occult	-	-	-	-	-	-	-	-	-	-
Others	-	-	-	-	-	-	-	-	-	-
Status of the fellow eye										
Initial/intermediate AMD	-	-	-	-	-	-	-	-	-	-
Neovascular	-	-	-	-	-	-	-	-	-	-
Atrophic	-	-	-	-	-	-	-	-	-	-
Disciform scar	-	-	-	-	-	-	-	-	-	-
Others	-	-	-	-	-	-	-	-	-	-
Status of the lens										
Cataract	-	-	-	-	-	-	-	-	-	-
Pseudophakia	-	-	-	-	-	-	-	-	-	-
Transparent	-	-	-	-	-	-	-	-	-	-
BCVA	153	0.6119	0.3314	1.1298	0.1164	153	*0.313*	*0.1742*	*0.5623*	*0.0001*
CMT change	151	1.234	0.5158	2.9522	0.6367	151	0.5479	0.2503	1.1994	0.1323
OCT assessment										
SRF	43 (29.25)	0.9004	0.4568	1.9927	0.9004	24 (17.02)	1.0262	0.421	2.5014	0.9547
Thickening	9 (6.12)	0.4934	0.1414	2.5674	0.4934	4 (2.84)	-	-	-	0.9891
Both	9 (6.12)	0.2342	0.5657	10.2697	0.2342	9 (6.38)	1.516	0.3851	5.9677	0.5518
None	86 (58.5)	1	-	-	-	104 (73.76)	1	-	-	-
Macular associated lesions										
Bleeding >50%	1 (0.9)	-	-	-	0.9944	1 (0.88)	-	-	-	0.9944
PED	16 (14.41)	0.7143	0.2001	2.5495	0.6042	15 (13.16)	0.4667	0.1135	1.9195	0.2909
RPE tear	3 (2.7)	-	-	-	0.9907	2 (1.75)	-	-	-	0.9924
Initial minimal atrophy	20 (18.02)	2.1429	0.5856	7.8414	0.2495	26 (22.81)	*5.3667*	*1.1472*	*25.105*	*0.0328*
Initial minimal fibrosis	47 (42.34)	*0.1931*	*0.0662*	*0.5632*	*0.0026*	52 (45.61)	*0.1273*	*0.0374*	*0.4332*	*0.001*
RAP	0 (0)	-	-	-	-	0 (0)	-	-	-	-
PV	0 (0)	-	-	-	-	1 (0.88)	-	-	-	0.9946
Others	24 (21.62)	1	-	-	-	17 (14.91)	1	-	-	-
Total time of treatment	-	-	-	-	-	-	-	-	-	-
Number of injections	153	1.1762	0.9157	1.5107	0.2038	150	1.0851	0.8399	1.4021	0.5319
Number of visits	146	1.1098	0.9481	1.2991	0.1949	146	0.9166	0.7719	1.0885	0.3207
	3 years					4 years				
	n (%)	OR	CI 95%		*p*-value	n (%)	OR	CI 95%		*p*-value
Age	-	-	-	-	-	-	-	-	-	-
Sex										
Female	-	-	-	-	-	-	-	-	-	-
Male	-	-	-	-	-	-	-	-	-	-
Year of diagnosis										
2007	-	-	-	-	-	-	-	-	-	-
2008	-	-	-	-	-	-	-	-	-	-
2009	-	-	-	-	-	-	-	-	-	-
2010	-	-	-	-	-	-	-	-	-	-
Delay of treatment										
< 30 days	-	-	-	-	-	-	-	-	-	-
(30–90] days	-	-	-	-	-	-	-	-	-	-
> 90 days	-	-	-	-	-	-	-	-	-	-
Angiographic type of lesion										
Classic	-	-	-	-	-	-	-	-	-	-
Predominantly classic	-	-	-	-	-	-	-	-	-	-
Minimally classic	-	-	-	-	-	-	-	-	-	-
Occult	-	-	-	-	-	-	-	-	-	-
Others	-	-	-	-	-	-	-	-	-	-
Status of the fellow eye										
Initial/intermediate AMD	-	-	-	-	-	30 (20.83)	1	-	-	-
Neovascular	-	-	-	-	-	20 (13.89)	0.6667	0.212	2.0963	0.4879
Atrophic	-	-	-	-	-	46 (31.94)	0.84	0.3343	2.1105	0.7107
Disciform scar	-	-	-	-	-	35 (24.31)	1.0588	0.3992	2.8085	0.9086
Others	-	-	-	-	-	13 (9.03)	0.4444	0.112	1.7634	0.2488
Status of the lens										
Cataract	-	-	-	-	-	-	-	-	-	-
Pseudophakia	-	-	-	-	-	-	-	-	-	-
Transparent	-	-	-	-	-	-	-	-	-	-
BCVA	153	*0.2781*	*0.1539*	*0.5026*	*<0.0001*	153	*0.3332*	*0.1982*	*0.56*	*<0.0001*
CMT change	151	*0.4459*	*0.2122*	*0.937*	*0.003*	151	*0.4485*	*0.226*	*0.8901*	*0.0219*
OCT assessment										
SRF	15 (15)	1.1722	0.3878	3.543	0.7784	5 (10)	2.8	0.4205	18.644	0.2871
Thickening	4 (4)	0.3419	0.0341	3.4296	0.3616	0 (0)	-	-	-	-
Both	2 (2)	-	-	-	0.988	2 (4)	-	-	-	-
None	79 (79)	1	-	-	-	43 (86)	1	-	-	-
Macular associated lesions										
Bleeding >50%	0 (0)	-	-	-	-	0 (0)	-	-	-	-
PED	8 (8.89)	0.5	0.065	3.8453	0.5054	1 (2.13)	-	-	-	0.9947
RPE tear	3 (3.33)	-	-	-	0.9902	1 (2.13)	-	-	-	0.9943
Initial minimal atrophy	26 (28.89)	*6.3*	*1.2752*	*31.1244*	*0.0239*	12 (25.53)	6	0.3901	92.277	0.1988
Initial minimal fibrosis	43 (47.78)	0.3971	0.0919	1.7149	0.2159	30 (63.83)	0.4	0.0301	5.3073	0.4873
RAP	0 (0)	-	-	-	-	0 (0)	-	-	-	-
PV	0 (0)	-	-	-	-	0 (0)	-	-	-	-
Others	10 (11.11)	1	-	-	-	3 (6.38)	1	-	-	-
Total time of treatment	-	-	-	-	-	153	0.9844	0.9557	1.014	0.2991
Number of injections	114	*1.4048*	*1.081*	*1.8254*	*0.011*	55	*1.3943*	*1.01*	*1.9249*	*0.0434*
Number of visits	114	0.9166	0.7719	1.0885	0.3207	58	1.1477	0.9012	1.4617	0.2642

A total of 153 patients were included in the final univariate analysis for presence of geographic atrophy vs. fibrotic scar. This table shows all the variables evaluated in each visit during the 4 years of follow-up. OR: odds ratio, CI: confidence interval, AMD: age-related macular degeneration, BCVA: best corrected visual acuity, CMT: central macular thickness, SRF: subretinal fluid, PED: pigment epithelium detachment, RPE: retinal pigment epithelium, RAP: retinal angiomatous proliferation, PV: polypoidal vasculopathy. Statistically significant results appear in italic

Regarding univariate analysis results, statistically significant factors associated with higher probability of GA were: small area of atrophy (not involving the fovea nor being the main component of lesion) at 2 and 3 years of follow-up, and the greater number of injections at 3 months, 3 and 4 years. On the other hand, negative predictors of GA, were male sex, lower vision at 2, 3 and 4 years of follow-up, the lower change in central macular thickness at 2, 3 and 4 years, and presence of a small area of fibrosis (not involving the fovea nor being the main component of lesion) at 3 months, 1 and 2 years of follow-up.

**Fig. 2:**
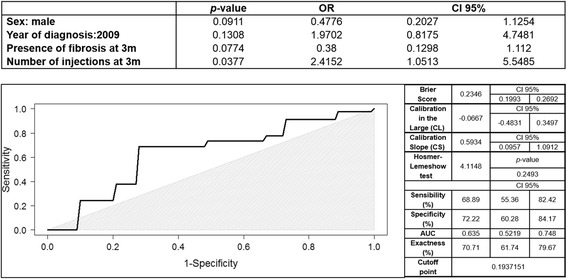
Multivariate analysis and predictive model Receiver Operating Characteristic (ROC) curve of geographic atrophy vs. fibrotic scar. The upper chart shows results of multivariate analysis. The figure below shows ROC curve of the predictive model. The area under the curve reveals that this model has moderate reliability in the prediction of final macular anatomic status. The greyish area indicates where the model loses its reliability. Besides, on the right internal validation data is shown. CI: confidence interval, AUC: area under the curve, CL: calibration in the large, CS: calibration slope

Multivariate analysis (Fig. 2) to find the best predictive model was performed using those previous variables with *p* < 0.1. Measurements after the first year of follow-up were excluded, as they would have low predictive value. As a result, sex, diagnosis in 2009, presence of a small area of fibrosis at 3 months, and number of injections at 3 months were chosen, and the best-fitted model (Fig. 2) included the 4 of them.

## Discussion

In this study, we identify potential predictors and introduce new predictive models of final anatomic outcome in neovascular AMD treated with as-needed ranibizumab. Previous models aim to identify patients at early or intermediate stages at high risk of advanced AMD, and many of them include a combination of demographic, clinical, genetic and environmental variables [19–21]. We propose two predictive models of anatomic outcome based on selected demographic and clinical features easily harvested in the daily clinical routine. Descriptive results of this sample have been previously published [8].

We assessed presence of GA by analysing colour fundus photographs, fundus autofluorescence and OCT. In our sample, 37.11% of patients with inactive macular status showed GA at the end of the follow-up. Comparison of Age-Related Macular Degeneration Treatments Trials (CATT) study reported new GA lesions in 12.9–25.8% of patients with no GA at enrolment after 2 years of follow-up, in patients randomly assigned to injections of ranibizumab or bevacizumab and to a 2-year dosing regimen of monthly or PRN or to monthly for 1 year and PRN the following year [6]. Independent baseline risk factors found in CATT for GA development included poor BCVA, RAP, foveal intraretinal fluid, monthly dosing, and treatment with ranibizumab [6]. Thus, anti-VEGF therapy was suggested to play a role in GA development.

In the present study, we included patients who showed a small area of atrophy at baseline, not involving the fovea nor being the main component of lesion, but we did not find baseline atrophy associated with further development of GA. However, presence of atrophy at 2 and 3 years of follow-up was associated with higher probability of GA as final macular status. Also, the greater number of injections was associated with GA, and this fact reinforces the association of GA with anti-VEGF therapy previously proposed. Interestingly, GA development has only been observed during the treatment of AMD, in which GA is part of the natural history of the disease, and has not been observed during the course of other diseases managed by multiple injections of anti-VEGF drugs. Furthermore, the study of growth of GA performed by CATT showed that eyes with GA farther from the fovea, which we included in the present study, had higher growth rates by 0.14 mm/year for every millimetre farther from the fovea [22]. This could explain the fact that, in our sample, we found a significant association between macular atrophy and final GA, and may represent the evolution of the disease.

Another recent study, in which RPE atrophy was monitored using polarization-sensitive OCT, revealed an increase in atrophic RPE features and GA dimension on 61% of patients at 2 years of follow-up following a similar regimen of injections with ranibizumab [23]. Additionally, Tanaka et al. in a retrospective study, observed GA developing outside CNV margin only in those eyes that showed GA outside the lesion at baseline, suggesting that atrophic scars that mimic GA could emerge within the area originally occupied with a CNV [3]. In this study we included atrophic scars in the term GA, and they are referred to as GA throughout this report.

SEVEN-UP study enrolled patients from the ANCHOR and MARINA trials, that had received 2 years of monthly ranibizumab followed by an additional 2 years of as-needed ranibizumab treatment in the HORIZON protocol, and were recalled for evaluation at 7 to 8 years after their enrolment. In this study they detected macular atrophy by fundus autofluorescence analysis in 98% of study eyes, and 90% showed decreased autofluorescence involving the fovea at the end of the follow-up [24]. Predictors found to be associated with final atrophy at SEVEN-UP were baseline area of atrophy and baseline area of leaking CNV [25]. As evolution to atrophy can limit the result of treatment with anti-angiogenic drugs, further studies aiming to avoid this evolution will be needed. Our predictive models could help to selecting patients to be enrolled in such studies.

CATT study analysed the risk of scar development in a recent report [5]. Scars were classified as fibrotic and nonfibrotic attending to their characteristics observed at colour fundus photographs and FA. Fibrotic scars are relatively easy to recognize at ophthalmoscopy. On the contrary, as nonfibrotic scars are funduscopically identical to de novo GA, they were distinguished by FA [5]. As mentioned before, we included atrophic scars in the term GA, because, as this is an observational study and we didn’t perform FA at the end of the follow-up. CATT study reported 45.3% of scar development after 2 years of follow-up, and predictors for scar formation (either fibrotic or nonfibrotic) included classic CNV, blocked fluorescence on FA, increased retinal thickness, foveal subretinal fluid and dome-shaped subretinal hyperreflective material [5].

SEVEN-UP study reported 61.4% of the study eyes showing macular subretinal fibrosis and 38.6% of the eyes presenting fibrosis involving the foveal centre [24]. Given the absence of fibrotic scar in almost 40% of study eyes, it was hypothesized that anti-VEGF therapy may alter the natural course of neovascular AMD by prolonging the active phase of the disease by preserving outer retina and RPE [24]. In our sample, 41.75% of the eyes with inactive AMD at the end of the follow-up showed fibrotic scar, although follow-up period was shorter. Based on our clinical experience, fibrosis is a complication that appears earlier than atrophy, which explains that in our study we found similar percentages of fibrosis compared with SEVEN-UP, with longer follow-up.

We found transparent lens associated to preserved macular anatomy at the end of the follow-up, however, we rejected this as a valid predictor due to the fact that only 3 out of 131 studied eyes for this variable showed this condition.

Both predictive models showed AUC values significantly different from 0.5 and, consequently, are considered appropriate. Predictive model for GA vs. fibrotic scar showed an AUC = 0.635 and included diagnosis in 2009, presence of fibrosis at 3 months and number of injections at 3 months. Curiously, in the year 2009, we observed inappropriately low mean number of visits (5.37) and injections (0.86), so we decided investigate the year of diagnosis as a variable. As a result, diagnosis in 2009 was associated with less probability of preserved macula, although number of visits and number of injections were not identified as predictors of preserved macula at any time point. In this year, pro re nata treatment basis were being established [26, 27], and this fact may justify the low number of visits and injections, and may have interfered with our results. Furthermore, patients enrolled in this study showed better outcomes from the year 2010 on.

The predictive model of preserved macula vs. GA/fibrotic scar showed a greater AUC (0.76). Therefore, it was considered a more suitable model than the other, and included age, baseline BCVA, and number of injections at 3 months. This highlights these factors as important predictors of final macular status, and, according to our results, young patients, those with good baseline vision and those who receive a correct loading dose would have greater probability of well-preserved macula as final anatomic status. Besides, these anatomic predictors have been previously identified as visual predictors [28].

In the current study we find some limitations. First, as data were collected from medical charts, they might not contain all the information needed. Also, daily clinical routine does not allow a strict regimen of visits and treatment as performed in clinical trials, so that, variability could exist at this point. For this reason, we identified number of injections at 3 months (90 days) as a predictor, but this number should be the same (3) for all patients according to as-needed treatment protocol. All patients enrolled in our study had received loading dose in a reasonable period of time, however, as this study was performed on a daily clinical practice basis, the interval between injections was not as strict as in clinical trials (30 days), and this resulted in a mean number of injections at 3 months of 2.54 instead of 3. Besides, reproducibility of this study may be limited due to the characteristics of the design. Another important limitation was the high number of dropouts, mainly due to the fact that we excluded those patients who had not completed the follow-up period. Moreover, we excluded patients who did not respond to treatment, and this could have interfered with final outcomes. We did not exclude patients presenting a small area of atrophy or fibrosis at baseline, and development of GA or fibrotic scar could represent the progression of the disease. Finally, our predictive models are based on easily harvested clinical and demographic risk factors, and could be attractive and practical to apply in the clinical routine. However, the addition of other variables, such as genetic factors, which are more difficult to obtain, could increase the sensitivity and specificity of the models.

## Conclusions

We have identified predictors of final macular status, and additionally, based on these predictors we propose two predictive models. Predictive model of preserved macula should be validated in a prospective study with a different cohort of patients to be considered as a useful tool for individual patient management and clinical research studies.
